# Osteoradionecrosis of the Scalp: A Case Series Highlighting Clinical Challenges and Literature Gaps

**DOI:** 10.1002/oto2.70257

**Published:** 2026-07-01

**Authors:** Claire M. E. Gleadhill, Charles J. Gallego, Camila Hurtado, Troy J. Weinstein, Jared R. Robbins, Audrey H. Baker

**Affiliations:** ^1^ Department of Otolaryngology–Head & Neck Surgery University of Arizona Tucson Arizona USA; ^2^ College of Medicine – Tucson University of Arizona Tucson Arizona USA; ^3^ Department of Radiation Oncology University of Arizona Tucson Arizona USA; ^4^ Present address: Department of Otolaryngology–Head and Neck Surgery at Case Western Reserve University Cleveland Ohio USA; ^5^ Present address: Department of Radiation Oncology at Duke University Durham North Carolina USA

**Keywords:** calvarium, cutaneous carcinoma, osteoradionecrosis, scalp, skin cancer

## Abstract

**Objective:**

Cutaneous carcinoma of the scalp is a common, but underreported, and complications of scalp irradiation remain poorly described. Many patients receive primary or postoperative radiation to the scalp, and complications from treatment are not well described. Calvarial osteoradionecrosis can be difficult to treat, and it is imperative that research be done to learn more about this disease. In this case series, we attempt to define its incidence and identify risk factors for the development of osteoradionecrosis of the calvarium.

**Study Design:**

Retrospective study.

**Setting:**

Single tertiary academic medical center.

**Methods:**

Information, including diagnosis, surgery, radiation dose, past medical history, time to bone exposure, and treatment, was analyzed for all patients undergoing scalp irradiation from 2018 to 2023.

**Results:**

Forty‐five patients received scalp irradiation for cutaneous head and neck cancer, and 14 developed calvarial osteoradionecrosis. Ten patients had both their original surgery and radiation at our institution, yielding an institutional incidence of 24.4% of calvarial osteoradionecrosis. All patients who developed calvarial osteoradionecrosis originally underwent surgery followed by postoperative radiation, with the median amount of time between surgery and radiation being 42 days (36‐114 days) and a median radiation dose of 57.5 grays in 25 fractions. The average time between completion of radiation therapy and development of osteoradionecrosis was 890 days (2.4 years). All patients were managed conservatively without surgical intervention, hospitalization, or intracranial complications.

**Conclusion:**

Osteoradionecrosis of the calvarium is an underreported late complication of scalp radiation that can be managed conservatively.

Cutaneous carcinoma of the head and neck is among the most prevalent cancers worldwide.^1^, ^2^ While the scalp accounts for approximately 3% to 20% of these malignancies, it remains underrepresented in the literature.^1^, ^2^, ^3^ The standard of care for cutaneous carcinoma of the head and neck typically involves surgery and radiation therapy (RT).^1^ The complications of radiation to other sites of the body have been well documented in existing research,^4^, ^5^ yet major radiation‐associated complications specific to the scalp—particularly calvarial osteoradionecrosis (ORN)—remain largely unexamined.^5^

ORN occurs when irradiated bone becomes necrotic due to radiation‐induced vascular damage and compromised blood supply.^6^ It is defined by the presence of non‐healing, exposed, irradiated bone in a wound persisting for more than 3 months.^7^ The severity of ORN varies widely, ranging from small wounds that respond to conservative management to extensive necrosis with significant bone exposure.^8^ In fact, a calvarial ORN grading system was developed by Weerakkody et al that staged ORN into three stages depending on the amount of exposed calvarium, evidence of ORN on imaging, and the presence of intracranial infections.^9^ Although ORN of the calvarium has been studied in a limited fashion, ORN of the mandible has been extensively investigated. According to O'Dell and Sinha, ORN typically develops between 22 and 47 months after completion of RT.^10^ Additionally, mandibular ORN is most commonly observed in patients who have received high radiation doses, often exceeding 60 Gy.^11^ However, no established dose thresholds exist for calvarial ORN. Due to its anatomical location and complex nature, ORN of the calvarium presents significant treatment challenges.

There are a limited number of studies that have addressed calvarial ORN, highlighting a substantial gap in understanding this disease entity. This retrospective study aims to characterize the clinical presentation of calvarial ORN following radiation, report its incidence within this cohort, and qualitatively assess risk factors, complications, and patient outcomes.

## Methods and Materials

### Study Design, Inclusion/Exclusion Criteria, and Nomenclature

A retrospective chart review was conducted on all patients diagnosed with head and neck cancer of the scalp who underwent RT at a single tertiary academic center between 2018 and 2023. Patients were included if they had a confirmed diagnosis of head and neck cancer of the scalp—including squamous cell carcinoma (SCC), basal cell carcinoma (BCC), melanoma, and other cutaneous malignancies—and received RT to the scalp, regardless of whether they subsequently developed ORN. For this study, ORN was defined as exposed or necrotic bone identified clinically by the presence of exposed bone for greater than 3 months, determined by a head and neck surgical oncologic and reconstructive surgeon. The patients were then graded on their stage of ORN based on the staging system proposed by Weerakkody et al.^9^ In this staging system, stage I was classified as patients with exposed and intact calvarium with soft tissue ulceration; stage II was classified as patients with calvarial destruction identified on imaging; and stage III was classified as patients with intracranial infection and full‐thickness bony destruction. Patients were excluded if they had insufficient clinical documentation. Successful management of ORN was defined as the absence of severe complications such as meningitis, dural abscess, or encephalitis, along with no additional surgical intervention or hospitalization.

### Data Collection and Management

Patient data were extracted from the electronic medical record under the direct supervision of the primary investigator. Demographic variables, including age and sex, were collected, alongside relevant medical and social history, including comorbidities, medication use, smoking history, and alcohol consumption. Detailed information on the initial cancer diagnosis, treatment, and outcomes was obtained, including surgical intervention, radiation dose and duration, tumor characteristics from pathology reports, and post‐treatment outcomes. For patients who developed ORN, data on scalp wound characteristics, complications, time to bone exposure, conservative management approaches, and medical and surgical interventions.

All patient data were securely stored in a REDCap database, with patient confidentiality maintained through de‐identification of data.

### Statistical Analysis and Study Limitations

Given the limited sample size, the final cohort was insufficient for robust statistical analysis. As a result, a descriptive analysis was conducted, focusing on key patient variables that may have influenced the development of ORN of the scalp. The analysis examined surgical procedures, as well as the relationship between radiation dose, delivery method, and ORN occurrence, as well as ORN‐related complications and treatment outcomes. Four patients who were diagnosed with ORN of the calvarium were originally treated and radiated at outside institutions; therefore, they have been removed from the incidence calculation, and some details of their radiation and reconstruction are unknown.

### Ethical Considerations

Prior to initiation, this study was approved by the University of Arizona Institutional Review Board (IRB), Tucson, Arizona, IRB #STUDY00001843.

## Results

### Study Grouping and Baseline Characteristics

A total of 45 patients with cutaneous cancer of the scalp who received RT were included in the study. Patients were divided into two cohorts: cohort 1 (31 patients, 68.9%), who did not develop ORN of the calvarium, and cohort 2 (14 patients total, 31.1%; 10 originally treated at our institution, 24.4%), who progressed to develop ORN of the calvarium (see Table 1).

**Table 1:** Cohort Characteristics for Patients With and Without Osteoradionecrosis (ORN) of the Calvarium

		Total	Development of ORN			
		45	No (n = 31)		Yes (n = 14)	
			Mean	N	Mean	N
Age, y			78		77	
Gender	Male	39 (87%)		26 (84%)		13 (93%)
	Female	6 (13%)		5 (16%)		1 (7%)
Diabetes mellitus		7 (16%)		5 (16%)		2 (14%)
Immunocompromised		13 (29%)		10 (32%)		3 (21%)
Vascular disease		32 (71%)		24 (77%)		8 (57%)
Thyroid disease		8 (18%)		7 (23%)		1 (7%)
BMI			26.4		26.69	
Obesity		9 (20%)		5 (16%)		4 (29%)
Smoking history		16 (36%)		9 (29%)		7 (50%)
Radiation type	Postoperative	42 (93%)		28 (90%)		14 (100%)
	Primary	1 (2%)		1 (3%)		0
	Unknown	2 (4%)		2		0
Time between surgery and radiation, d			57 (median: 57)		63 (median: 42)	
Radiation dose, Gy			52 (median: 55)		60 (median: 57.5)	
Fractions			21		25	
Procedure	Mohs	15 (33%)		12 (39%)		3 (21%)
	WLE	28 (62%)		19 (61%)		9 (64%)
	Periosteal stripping	8 (18%)		4 (13%)		4 (29%)
	Bone drilling	3 (7%)		1 (3%)		2 (14%)
	Craniectomy	2 (4%)		0		2 (14%)
Reconstruction	None/unknown	5 (11%)		2 (6%)		3 (21%)
	Primary	17 (38%)		12 (39%)		5 (36%)
	Secondary	15 (33%)		11 (35%)		4 (29%)
	Secondary + integra + STSG	7 (16%)		5 (16%)		2 (14%)
	Free flap	1 (2%)		1 (3%)		0

Abbreviation: BMI, body mass index; STSG, split‐thickness skin graft; WLE, wide local excision.

The average age in cohort 1 was 78 years, with 26 males and 5 females. Among these patients, 5 had diabetes, 10 were immunocompromised, and 24 had vascular disease. The average body mass index (BMI) in this group was 26.4, and nine patients had a history of smoking. Treatment consisted of the following: all patients underwent surgery prior to radiation, including 15 who had Mohs surgery, 28 who underwent wide local excision—including 8 with periosteal stripping and 3 with bone drilling, and 2 who had a craniotomy. In terms of surgical reconstruction, 12 had their surgical sites primarily approximated, 11 had their wounds heal by secondary intention, 5 wounds healed by secondary intention with Integra and skin graft, and 1 patient had a free flap reconstruction. One patient was treated with primary RT. This cohort received a median radiation dose of 55 Gy in 21 fractions, with an average interval of 57 days between surgery and radiation. The timing of radiation treatment was unknown for two patients.

In cohort 2, the average age was 77 years, with 13 males and 1 female. Among these patients, two had diabetes, three were immunocompromised, and eight had vascular disease. The average BMI was 26.69, and seven patients reported a history of smoking. Four patients were treated at outside institutions and presented with ORN of the scalp; therefore, they were removed from the incidence calculation, and some details of their radiation and reconstruction are unknown. ORN involved the vertex scalp (7/14, 50%), frontal scalp (2/14, 14.3%), parietal scalp (2/14, 14.3%), superior forehead (1/14, 7.1%), temporal scalp (1/14, 7.1%), and anterior skull (1/14, 7.1%). All patients in this cohort underwent surgery prior to radiation, with three receiving Mohs surgery, nine undergoing wide local excision—including four with periosteal stripping and two with bone drilling, two requiring a craniotomy, and one undergoing a parotidectomy. In terms of surgical reconstruction, five had their surgical sites primarily approximated, four had their wound heal by secondary intention, two wounds healed by secondary intention with Integra and a skin graft, and three patients had an unknown prior reconstruction. This cohort received a median radiation dose of 57.5 Gy in 25 fractions, with a median interval of 42 days between surgery and radiation. The average time from radiation treatment to the development of ORN of the calvarium was 890 days (2.4 years). Seven patients (50%) were diagnosed with stage I ORN, 7 patients (50%) were diagnosed with stage II ORN, and zero patients were diagnosed with stage III ORN according to the ORN grading systems proposed by Weerakkody et al.^9^

### Complications and Treatment of Calvarial ORN

Of the patients diagnosed with calvarial ORN, the most common wound complications in cohort 2 were bleeding (5/14, 35.7%) and superficial infection (4/14, 28.6%). Notably, none of the patients developed meningitis or intracranial complications during the average follow‐up period of 13 months.

Most patients were managed conservatively. Half of the patients (7/14) used only Vaseline or Aquaphor as needed around the wound edges, while 2 patients (14.3%) required routine debridement in the clinic. Three patients (21.4%) reported not using any form of conservative wound care. In terms of medical management, 4 patients (28.6%) were treated with antibiotics, and 2 patients (14.3%) underwent hyperbaric oxygen therapy. While bony exposure persisted, conservative treatments were used to prevent wound progression and maintain patient comfort. None of the patients required surgical intervention or hospitalization for scalp ORN as their wounds remained stable. The management approach did not differ across ORN stages. Two patients with ORN (14.3%) developed a local recurrence of their primary malignancy, compared to six patients without ORN (19.4%). The overall local recurrence rate across cohorts was 18%.

## Discussion

RT is well known for its potential complications, including ORN.^5^, ^6^, ^7^, ^8^ While ORN of the mandible has been extensively studied and well documented in the literature,^10^ there is a notable lack of research on ORN of the calvarium. Cutaneous carcinoma of the head and neck is among the most prevalent cancers worldwide, and the scalp accounts for approximately 3% to 20% of these malignancies that are typically treated with surgery and radiation.^1^, ^2^, ^3^ This study presents a case series of calvarial ORN following RT for cutaneous malignancies of the scalp, aiming to provide insight into this underreported late complication.

Several risk factors, including smoking, prior surgery, and obesity, have been well established for the development of mandibular ORN.^12^, ^13^ However, no specific risk factors have been identified for calvarial ORN. Due to the small sample size of our study, no statistically significant correlations could be established regarding risk factors for ORN of the calvarium, a finding consistent with Weerakkody et al.^9^ Despite the small sample size and lack of statistical significance, we observed a trend toward higher rates of ORN in patients with a smoking history and those who underwent periosteal stripping. Half of the patients in cohort 2 (ORN) had a history of smoking, while only 29% in cohort 1 (no ORN) had a history of smoking. Similarly, 29% of patients in cohort 2 had periosteal stripping, compared to 13% in cohort 1. These trends may highlight the role of impaired wound healing and tissue vulnerability in the development of ORN. Larger, multi‐institutional studies are needed to further explore potential risk factors and identify preventive measures for this rare but significant complication.

An interesting finding in this study was that all patients who developed calvarial ORN had undergone primary surgical treatment prior to RT. While surgery is the standard first‐line treatment for most cutaneous scalp malignancies, it is also a well‐documented risk factor for ORN.^1^, ^14^, ^15^ Surgical intervention disrupts the vascular supply and lymphatic drainage of the tissue, which may increase susceptibility to radiation‐induced necrosis.

Conversely, radiation alone is typically reserved for patients who decline surgery or are poor surgical candidates; this gap may reflect a difference in baseline morbidity between our patient populations.^15^ Unfortunately, patients with indications for definitive or adjuvant radiation usually include those with more aggressive tumors, larger surgical wounds, deeper scalp involvement, and slow wound healing. Likewise, the postoperative radiation volume, total radiation dose, dose per fraction, and radiation technique may also contribute to the ORN risk in any given patient. Balancing the risk of recurrence and the risks of significant toxicity, like ORN, is a key determination in developing the treatment plan. Given the potential risk, patients receiving adjuvant RT after surgery should be closely monitored not only for cancer recurrence but also for early signs of ORN. Multidisciplinary care is essential to ensure these patients receive appropriate counseling on the risks of surgery and radiation while optimizing treatment strategies.

At our institution, the incidence of calvarial ORN was 24.4%, with a median radiation dose of 57 Gy in 25 fractions. In contrast, Weerakkody et al reported a much lower incidence (6.67%) in a cohort of 84 patients with cutaneous scalp malignancies, which is more consistent with the 5% incidence reported for mandibular ORN.^9^, ^10^, ^16^ Interestingly, in our cohort, 42 patients (93%) were treated with surgery prior to their radiation, while, in the study by Weerakkody et al, approximately 63 patients (approx. 75%) were treated with surgery prior to radiation.

Nonetheless, this difference in ORN rates could highlight the possibility that surgery may influence ORN by disrupting blood flow and the ability to heal wounds. Many of the patients in our study had surgical sites that were allowed to heal by secondary intention with or without a skin graft—51% in cohort 1 (no ORN) and 43% in cohort 2 (patients who developed ORN). While there were no statistically significant differences in the type of reconstruction and the development of ORN in our cohorts, none of the patients in the Weerakkody et al surgical sites were allowed to heal by secondary intention only. They found that surgery with skin grafting (compared to local tissue rearrangement and free flap reconstruction) was a significant risk factor in developing ORN.^9^ Perhaps allowing the wounds to heal by secondary intention only permits granulation tissue and epidermis to grow over the bony calvarium, but fails to provide a sufficient barrier or blood supply to the underlying tissue. When an additional dose of radiation is applied, the area may be at greater risk of developing ORN. More studies need to be done to determine if the type of surgical reconstruction is truly a risk factor for calvarial ORN.

Selection bias may have also had a role as our original calculated incidence was almost 32% when compared to Weerakkody et al's incidence of 6.67%. Four patients in our ORN‐positive cohort presented to our institution with already known ORN of the scalp, seeking treatment from the senior author. However, these patients were not diagnosed or radiated at our institution. These patients were included in our study to examine the treatment response characteristics of ORN. When these four patients are removed from the equation, our incidence dropped to 24.4%. This is still higher than the Weerakkody paper and highlights the fact that selection bias still may be playing a role. For example, patients who have been treated for chronic ORN wounds may be more likely to have been included in the data than patients who received radiation and did not have problems or who were lost to follow‐up, and so forth. Additionally, 50% of patients with ORN in our cohort were stage I, in comparison with only 16.7% of patients in the Weerakkody paper. Differences in the incidence of stage I ORN between our studies may reflect the fact that none of the patients in the Weerakkody paper healed by secondary intent. Wounds healing by secondary intent may predispose to mild ORN. Furthermore, variations in reporting practices, diagnostic thresholds, and frequency of follow‐up can also contribute to identifying milder forms of ORN. In addition, the small size of the cohort increases the impact of small variations. Multiple factors likely contribute to the high incidence of ORN observed in this study, including underlying patient risk factors. This paper is limited by a small sample size and the ability to find statistically significant correlations unique to this geographic region, such as environmental exposures, past medical history, and smoking history, that could also account for a high incidence rate of ORN.

Despite differences in incidence, the timeline of ORN development was similar, with our study showing an average onset at 29 months (890 days) post‐radiation, closely mirroring Weerakkody et al's finding of 28 months (approximately 852 days). Comparatively, mandibular ORN has been reported to develop between 22 and 47 months post‐radiation.^9^, ^10^, ^16^ This highlights the possibility that calvaria ORN may be vastly underreported in the literature as this is a late complication, and many patients may no longer be followed by their radiation oncologists this far out after their radiation treatment.

Surgical intervention has been proposed as a treatment strategy for calvarial ORN, particularly for larger, non‐healing wounds.^3^, ^9^, ^17^ In such cases, management may involve craniotomy with debridement of necrotic bone, calvarial reconstruction, and free flap coverage. Some argue that extensive surgical intervention is necessary due to the high failure rate of conservative treatment,^15^ yet this approach poses significant risks, including prolonged recovery and potential morbidity—factors that many patients may be unwilling or unable to endure. Furthermore, patients with ORN already have compromised tissue with limited healing capacity, making them suboptimal candidates for aggressive surgical intervention.^18^ Patel et al reported successful outcomes, defined by near complete healing of the wound and the absence of severe complications such as meningitis, dural abscess, or encephalitis, with nonoperative management of calvarial ORN.^19^ This is further supported by a recent systematic review by Pang et al, in which the authors emphasized the variability in treatment strategies for calvarial ORN and additionally noted that conservative management is often successful.^20^ While calvarial ORN is difficult to treat due to its chronic nature and associated discomfort, our findings align with Patel et al and Pang et al, suggesting that conservative wound care and close follow‐up may serve as a reasonable and safe treatment strategy for patients who are not surgical candidates.^19^, ^20^ Thus, it is possible that the treatment strategy for patients with this disease entity may be based on surgeon preference and experience. While no patients in our study experienced intracranial complications such as infection, close monitoring is essential, and patients should be advised to seek immediate medical attention for any changes in their wound or altered mental status.^21^

Despite these insights, our study has several limitations. As a retrospective, single‐institution analysis, it is susceptible to selection bias and limited generalizability. Treatment approaches for cutaneous scalp malignancies and their complications may differ across institutions, and our sample size of 45 patients, with only 14 developing ORN, restricted the ability to perform statistical analyses. These factors may limit the applicability of our findings to broader patient populations.

Calvarial ORN is a difficult problem to treat, and examining the causes behind it can help shape ideas to prevent it. Perhaps if the head and neck oncologic surgeon, who knows the patient will likely need radiation after surgery, considers local tissue rearrangement or free flap reconstruction at the time of the original surgery, it could help minimize the risk of ORN. Other ideas proposed to reduce the risk of ORN could include limiting radiation hotspots, smoking cessation, and perhaps neoadjuvant immunotherapy, such as cemiplimab.

When used as adjuvant therapy, cemiplimab has shown longer disease‐free survival in patients with high‐risk cutaneous SCC.^22^ Furthermore, a study by Gross et al showed a complete pathological response rate of 51% among patients with stage II to IV cutaneous SCC when used as neoadjuvant therapy.^23^ These findings highlight the potential role of neoadjuvant immunotherapy in de‐escalating postoperative radiation in select patients. While none of the patients in this case series received neoadjuvant immunotherapy, its use is increasing across practices. However, further research is needed to examine these potential implications.

Given the limited existing literature on calvarial ORN, further research is critical to establish standardized management guidelines and to optimize patient care. Prospective, multicenter studies are necessary to validate these findings, identify risk factors, and develop protective strategies. Additionally, advancements in radiation delivery techniques and wound care protocols may further reduce the incidence and severity of ORN, ultimately improving outcomes for patients undergoing RT for scalp malignancies.

## Conclusion

Cutaneous cancers of the scalp are common and often require RT, which carries the risk of ORN of the calvarium. This study presents a case series of 45 patients who received treatment and radiation of the calvarium with 14 patients who developed calvarial ORN. This is the largest case series studied in the United States and the second largest in the world. None of the patients studied underwent surgical intervention for ORN, and all had stable wounds with exposed bone, which were successfully managed with conservative treatment. Notably, no patients developed intracranial complications during follow‐up. This case series underscores the need for larger, multi‐institutional studies to establish standard‐of‐care guidelines for the management of calvarial ORN. Further research is essential to identify potential risk factors and develop preventive strategies to mitigate the occurrence of this challenging complication.

## Author Contributions

**Claire M. E. Gleadhill**: Organized database, led data collection, and drafted the manuscript. **Charles J. Gallego**: Assisted with data collection, analysis, and manuscript writing. **Camila Hurtado**: Contributed to data interpretation, manuscript writing, editing, and formatting. **Troy J. Weinstein**: Contributed to manuscript writing, editing, and formatting. **Jared R. Robbins**: Contributed to project development, data analysis, and manuscript writing. **Audrey H. Baker**: Oversaw project development, data analysis, and manuscript writing.

## Disclosures

### Competing interests

The authors declare no conflicts of interest.

### Funding source

No funding or financial support was received for this study.
